# Electrical Wiring of the Aldehyde Oxidoreductase PaoABC with a Polymer Containing Osmium Redox Centers: Biosensors for Benzaldehyde and GABA

**DOI:** 10.3390/bios4040403

**Published:** 2014-11-03

**Authors:** Artavazd Badalyan, Marlen Dierich, Konstanze Stiba, Viola Schwuchow, Silke Leimkühler, Ulla Wollenberger

**Affiliations:** Department of Molecular Enzymology, Institute for Biochemistry and Biology, University of Potsdam, Karl-Liebknecht-Str. 24-25, 14476 Potsdam (Golm), Germany

**Keywords:** redox polymer, aldehyde oxidoreductase, ionic strength, benzaldehyde, GABA, biosensor

## Abstract

Biosensors for the detection of benzaldehyde and γ−aminobutyric acid (GABA) are reported using aldehyde oxidoreductase PaoABC from *Escherichia coli* immobilized in a polymer containing bound low potential osmium redox complexes. The electrically connected enzyme already electrooxidizes benzaldehyde at potentials below −0.15 V (*vs*. Ag|AgCl, 1 M KCl). The pH-dependence of benzaldehyde oxidation can be strongly influenced by the ionic strength. The effect is similar with the soluble osmium redox complex and therefore indicates a clear electrostatic effect on the bioelectrocatalytic efficiency of PaoABC in the osmium containing redox polymer. At lower ionic strength, the pH-optimum is high and can be switched to low pH-values at high ionic strength. This offers biosensing at high and low pH-values. A “reagentless” biosensor has been formed with enzyme wired onto a screen-printed electrode in a flow cell device. The response time to addition of benzaldehyde is 30 s, and the measuring range is between 10–150 µM and the detection limit of 5 µM (signal to noise ratio 3:1) of benzaldehyde. The relative standard deviation in a series (n = 13) for 200 µM benzaldehyde is 1.9%. For the biosensor, a response to succinic semialdehyde was also identified. Based on this response and the ability to work at high pH a biosensor for GABA is proposed by coimmobilizing GABA-aminotransferase (GABA-T) and PaoABC in the osmium containing redox polymer.

## 1. Introduction

Recent investigations on the periplasmatic aldehyde oxidoreductase from *Escherichia coli* (PaoABC) showed great potential of this enzyme for bioanalytical application and development of bioelectronic devices [1,2,3]. In *E. coli*, this enzyme is involved in detoxification of aldehydes to their less toxic acids to avoid cell damage [1]. PaoABC exhibits high catalytic activity for oxidation of different aromatic aldehydes, which are oxidized to respective carboxylic acids. The net reaction can be described with the following equation:

R-CHO + H_2_O → R-COOH + 2 H^+^ + 2 e^−^

PaoABC is regenerated through the concomitant transfer of two electrons to a variety of synthetic electron acceptors. Despite the similarity to other molybdoenzymes of the xanthine oxidase family, PaoABC has a different pH-behaviour because it is active at acidic and basic conditions depending on the electron acceptor and ionic strength conditions [3]. Under steady state conditions, the turnover of PaoABC is maximal at pH 4 for ferricyanide and reaches the maximum above pH 9 with a cationic metal complex the [Os(N,Nʹ-dimethyl-2,2ʹ-biimidazole)_3_]^2+/3+^. Thus, with the right choice of electron acceptor, biosensor operating conditions may be tuned.

Benzaldehyde oxidation in the presence of ferricyanide as electron acceptor is very fast with this enzyme. Benzaldehyde is a reactive, toxic, skin irritant substance which is produced in the millions of pounds per year [4,5,6,7]. Recently, we developed a biosensor for determination of benzaldehyde in pharmaceutical formulas on the basis of PaoABC entrapped in a polyvinylalcohol hydrogel and ferricyanide mediator [2]. The sensitivity was comparable or better than that of the alternative methods for benzaldehyde measurement including HPLC/UV [4,7,8,9,10], SPR-immunosensor [11], chemiluminescence [12], and differential pulse voltammetry on mercury drop electrodes [5]. However, this biosensor operates at high working potential, where unspecific oxidations are possible, and comprises the experimental inconvenience to add mediator to the solution. These points can be improved by using immobilized low potential mediators.

Redox hydrogels based on poly(4-vinylpyridine) and poly(N-vinyl)imidazole functionalized with various Os-complexes comprise attractive properties for the electrical communication between oxidoreductases and conductive supports and as hydrophilic matrixes, in which enzymes get stabilized and the permeation of the substrate is rapid. They are therefore attractive for the development of electrochemical biosensors [13,14,15,16] and biofuel cells [17,18,19,20,21] as has been demonstrated already using a variety of different enzymes, among them glucose oxidase [13,17,22,23], glucose dehydrogenase [24], cellobiose dehydrogenase [25,26], pyranose dehydrogenase [16,25], laccase [17], bilirubin oxidase [19,20], and sulphite oxidase [14].

In this work, PaoABC was electrically wired in an electron-conducting redox hydrogel that contains the redox complex [Os(N,Nʹ-dimethyl-2,2ʹ-biimidazole)_3_]^2+/3+^. This redox polymer has a low potential and contains mobile 13-atom-long tethers between the cationic redox center and the polymer backbone which introduces high mobility for the electron shuttling in the swollen polymer [23]. A shift of the optimum operational pH for the biosensor is expected since PaoABC is oxidizing benzaldehyde with the soluble [Os(N,Nʹ-dimethyl-2,2ʹ-biimidazole)_3_]^2+/3+^ at highly basic conditions [3]. This will allow coupling of further enzymes to enlarge the spectrum of bioanalytic applications of immobilized PaoABC. Here, we describe a biosensor comprising PaoABC immobilized in such a redox polymer with particular attention to pH and ionic strength and demonstrate its potential for benzaldehyde biosensing in a flow-cell arrangement. In addition, due to the substrate spectrum and the ability to tune the pH-optimum a novel biosensor utilizing coimmobilized GABA-aminotransferase (GABA-T) and PaoABC for the determination of the neurotransmitter γ-aminobutyric acid (GABA) is also reported.

## 2. Experimental Section

### 2.1. Chemicals

Benzaldehyde, potassium ferricyanide, and citric acid-monohydrate were from Sigma-Aldrich (Deisenhofen, Germany). Poly (ethylene glycol) (400) diglycidyl ether (PEGDGE) was purchased from Polysciences (Warrington, PA, USA). Poly (4-vinylpyridine)-[osmium-(N,N′-methylated-2,2′-biimidalzole)_3_]^2+/3+^ (osmium containing polymer, Os-polymer) prepared according to a published procedure [23] was a generous gift from Lo Gorton (Lund University, Sweden).

Sodium hydrogen phosphate monohydrate, tris (hydroxymethyl) aminomethane (Tris) from Serva GmbH (Heidelberg, Germany) and sodium chloride from Roth (Karlsruhe, Germany) were used.

The buffers used were citrate-phosphate buffer (McIlvaine’s buffer with 0.2 M Na_2_HPO_4_ and 0.1 M citric acid) in the pH-range from 4.0–8.0 and 50 mM Tris, 0.1 M KCl at pH 7.0–9.0. For pH-dependence we used citrate-phosphate buffer (ionic strength 50 mM) in the pH-range 4–7 and Tris/KCl (ionic strength 50 mM) in the pH-range 7–9. The ionic strength of solutions was varied simply by addition of an appropriate amount of KCl.

All chemicals were of analytical grade and used without further purification. All solutions were prepared with deionized water (Millipore, Eschborn, Germany).

Aldehyde oxidoreductase from *E. coli* (PaoABC, activity 20 U·mg^−1^, 53 µM) was expressed and purified as described earlier [1]. The amount of active enzyme was determined to be of 49% of the total enzyme on the basis of the difference of the spectra after reduction with benzaldehyde (active enzyme) and with dithionite for the complete reduction (total enzyme). The activity of PaoABC was 200 U·mL^−1^ using benzaldehyde (0.5 mM) as a substrate and the electron acceptor ferricyanide (0.1 mM) in citrate-phosphate buffer pH 6. γ−Αminobutyrate-2-oxoglutarate transaminase (GABA-aminotransferase, GABA-T) from *E. coli* was isolated and purified with modifications described in the literature [27,28]. *E. coli* total DNA from strain MG1655 was used to amplify GABA-aminotransferase which allowed cloning into the expression vector pET15b (Invitrogen). The resulting plasmid was designated pKS3 and expresses GABA-T with an N-terminal His_6_-tag fusion. The plasmid was transformed into *E. coli* BL21(DE3) cells and His_6_-GABA-T was expressed 4 h after induction with 100 µM IPTG. The recombinant protein was purified by affinity chromatography using nickel-nitrilotriacetic acid (Ni-NTA) resin (QIAGEN, Valencia, CA). The enzyme concentration was determined to be 205 µM utilizing the spectrum at λ = 280 nm using an extinction coefficient of ε = 15,840 M^−1^·cm^−1^.

### 2.2. Apparatus and Procedure

The stationary electrochemical experiments were performed with a home-made three-electrode electrochemical cell with a volume of 1 mL placed in a Faraday cage. A platinum wire (diameter 0.5 mm, Goodfellow, Germany) was used as counter electrode and Ag|AgCl|KCl (1 M) reference electrode against which all potentials are reported. The working electrodes were of spectrographic graphite (custom made configuration from a 3 mm spectrographic graphite bar, Ringsdorff-Werke, Germany). Amperometric measurements were performed using the Biometra EP30 potentiostat (Göttingen, Germany). Voltammetric measurements were carried out with CH Instrument Model 440 (Austin, USA) and Gamry Reference 600TM potentiostat (Gamry, USA). Square Wave Voltammetry measurements were done with following parameters Esw = 50 mV, f = 50 Hz, ΔE = 4mV. Experiments under anaerobic conditions were performed in a glove box (Coy, USA) in the atmosphere of 2% H_2_ and 98% N_2_.

The flow experiments were carried out using a small custom made setup equipped with a screen-printed electrode composed of a 1 mm (diameter) carbon and Ag|AgCl electrode (Type: BST2-WE-C001, RE-Ag/AgCl, BST, Germany) described earlier in [14]. The measurements were performed using the Biometra EP30 potentiostat (Göttingen, Germany) and the currents were recorded with a DUO analog-digital converter (WPI, Berlin, Germany).

### 2.3. Electrode Modification

#### 2.3.1. Benzaldehyde Biosensor

The spectrographic graphite electrodes were polished on wet emery paper (P2000, Schröder, Germany) and washed thoroughly with Milli-Q water, sonicated for 2 min, and then rinsed with Milli-Q water and dried at 95 °C [29]. The pretreated electrodes were than modified with 5 µL of 54 µM PaoABC and 1 µL of a freshly prepared PEGDGE solution (2.5 mg·mL^−1^ in water) and after 5 min with 2.5 µL of osmium containing polymer solution (10 mg·mL^−1^ in water). For the modification of the screen-printed electrodes, the 1 mm (diameter) carbon working electrode was modified with 2.5 µL of 54 µM PaoABC, 2.5 µL of osmium containing polymer (10 mg·mL^−1^ in water) and 1 µL of a freshly prepared PEGDGE solution (2.5 mg·mL^−1^ in water). The freshly modified electrodes were put under vacuum for 25 min for complete cross-linking reaction before placing the electrode into the respective electrochemical cell. Electrodes prepared for later use were kept at 4 °C. The study of pH and ionic strength was performed in the following way. We started at pH 7. Then, the biosensor response was measured in three different (pH or I) solutions (three times for each curve). After this, the next measurement was made in standard buffer in order to have reference points in the course of the experiment.

#### 2.3.2. GABA-Biosensor

To fabricate the GABA-biosensor a premixed solution, including 2.5 µL of PaoABC (54 µM), 2.5 µL of GABA-T (205 µM) and 1 µL of a freshly prepared PEGDGE solution (2.5 mg·mL^−1^ in water), was placed on top of the polished end of the 3 mm (diameter) polished spectrographic graphite electrode. After 5 min, 2.5 µL of osmium containing polymer (10 mg·mL^−1^ in water) and 1 µL of a freshly prepared PEGDGE solution (2.5 mg·mL^−1^ in water) were spread on top of the first layer of the electrode and allowed to stand overnight (4 °C and constant humidity). Prepared electrodes were kept at 4 °C for later use.

## 3. Results and Discussion

### 3.1. PaoABC in Osmium Containing Polymer

**Figure 1:**
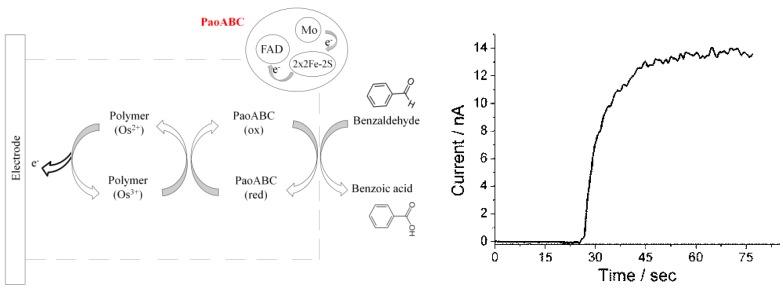
Illustration of the reaction sequence of the biosensor with PaoABC entrapped in osmium containing polymer on a spectroscopic graphite electrode and mediated electron transfer and (right) example of the steady state current response of the PaoABC/Os-polymer-sensor upon addition of benzaldehyde (5 µM in citrate-phosphate buffer pH 8) at the working potential +0 mV (*vs*. Ag|AgCl, 1 M KCl).

**Figure A1:**
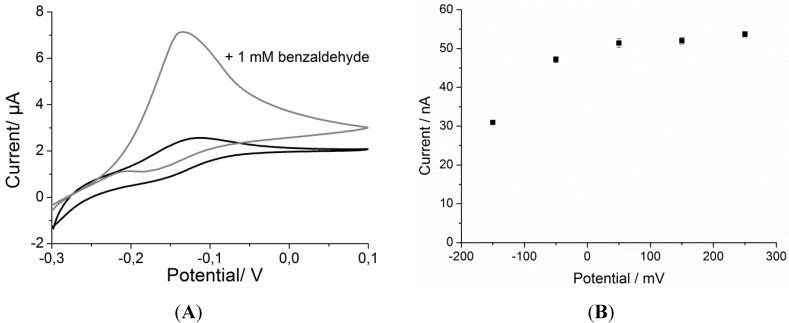
(**A**) Cyclic voltammogram of a PaoABC/Redox-polymer biosensor in 200 mM Tris-buffer pH 8.0 and after addition of 1 mM benzaldehyde. Scan rate 5 mV/s. (**B**) Amperometric responses of PaoABC/Redox-polymer biosensor to 20 µM benzaldehyde as a function of electrode potential, 100 mM phosphate buffer, pH 8.

PaoABC was immobilized on spectrographic graphite electrode in the redox polymer and covalently crosslinked by addition of PEGDGE to the mixture. In the absence of substrate, the midpoint of oxidation and reduction peaks of the bound osmium complexes can be seen in cyclic voltammetry at −0.182 V and catalytic enhancement in the presence of benzaldehyde (Appendix Figure A1A). The reaction sequence and a typical steady state response curve of the PaoABC biosensor for addition of 5 µM benzaldehyde is represented schematically in Figure 1. The addition of benzaldehyde resulted in the rapid appearance of an oxidation current at the PaoABC-biosensor when the electrode is polarized at 0 V. Benzaldehyde is oxidized to benzoic acid with concomitant reduction of the Moco-site of the enzyme. The reducing equivalents are then flowing from reduced catalytic Moco-site of PaoABC through a series of redox cofactors to osmium and further to the electrode. As a result, a catalytic oxidation current is generated proportional to benzaldehyde concentration with a lower limit of detection of 0.5 µM benzaldehyde. Blank experiments with the electrode modified with the polymer containing osmium complexes in the absence of enzyme showed almost no response (less than 1 nA) upon addition of 20 µM benzaldehyde. In order to find the optimum working potential, the oxidation currents towards the same concentration of benzaldehyde were measured at different potentials. The highest responses were obtained between −50 mV and 50 mV (Appendix Figure A1B). Therefore, 0 mV working potential was used for all further experiments.

**Figure 2:**
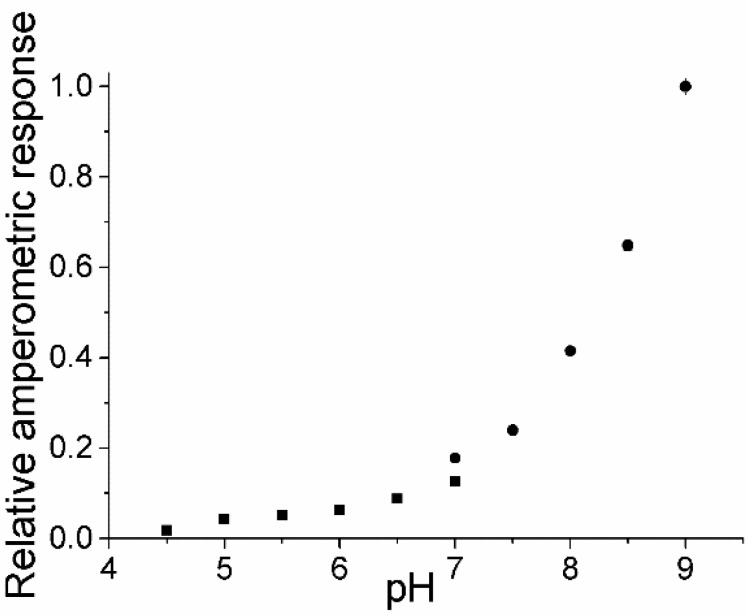
pH-dependence of amperometric response of PaoABC/Redoxpolymer-sensor towards 5 µM benzaldehyde, ■—in citrate-phosphate buffer pH 4–7, ●—in Tris buffer pH 7–9, working potential 0 mV (*vs*. Ag|AgCl|1 M KCl), n = 3.

**Figure A2:**
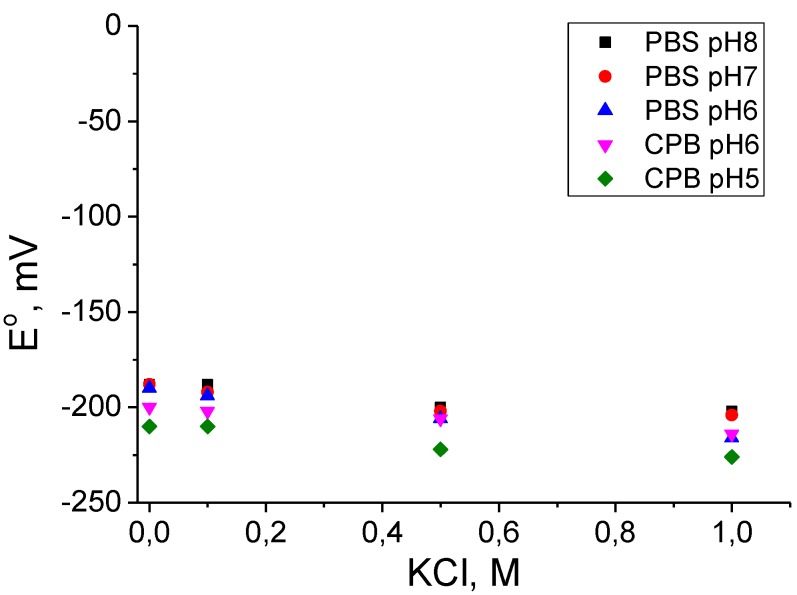
Formal potential E^0^ of osmium bound polymer of PaoABC/Redox-polymer biosensor as a function of KCl concentration at different pH values.

**Figure A3:**
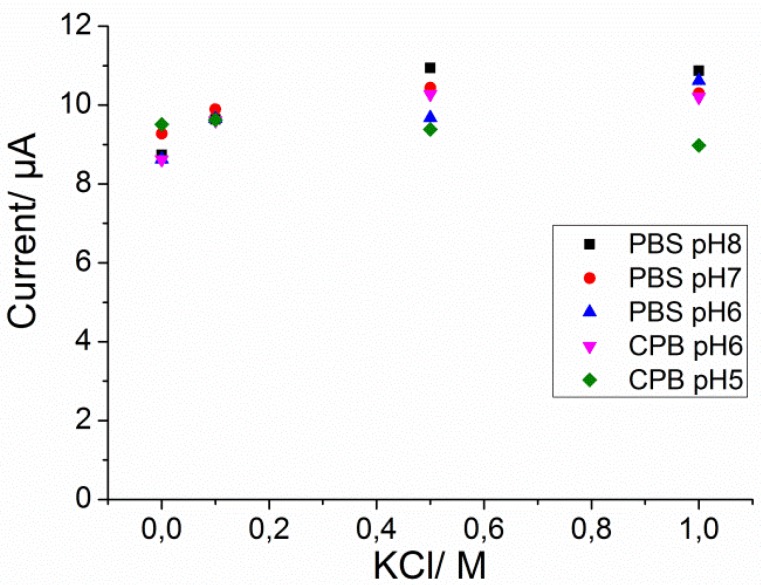
Anodic peak current of osmium containing polymer of PaoABC/Redox-polymer biosensor as a function of KCl concentration at different pH values.

**Figure A4:**
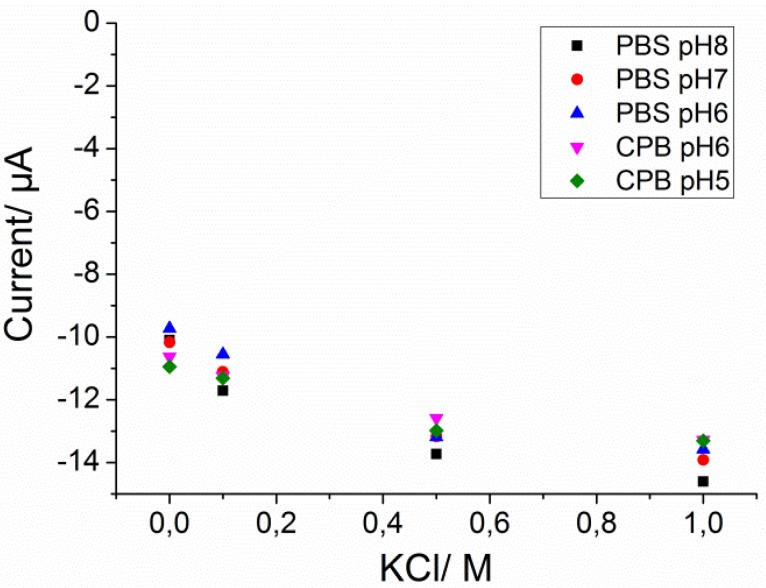
Cathodic peak current of osmium containing polymer of PaoABC/Redox-polymer biosensor in buffer solutions of different pH values and increasing KCl-concentration.

Figure 2 shows the pH-dependence of the catalytic current obtained for PaoABC in the osmium containing polymer determined by measuring the amperometric responses towards 5 µM benzaldehyde in the range of pH 4.5–9. The response is strongly pH-dependent. The highest signals with PaoABC in osmium containing redox polymer were obtained at pH 9. Lower pH diminishes the response and at pH 4.0 no response is recorded and the immobilized enzyme is irreversibly inactivated. The opposite behavior was observed for ferricyanide both in solution and with PaoABC immobilized in a polyvinylalcohol hydrogel [1,2]. The electrochemical properties of osmium containing polymer with PaoABC were also studied at different pH-values in the absence of substrate by using cyclic voltammetry evaluating the cathodic and anodic peak currents and the standard potential. The biosensor showed no dramatic change in the voltammograms with the variation of pH (Appendix Figure A2, Appendix Figure A3 and Appendix Figure A4). This underlines also that the pH profile is not a result of structural changes in the polymer and thus electron transfer capability.

The redox polymer consists of the polymeric backbone and covalently bound osmium complexes acting as mediators. In a recent work, the interaction between PaoABC and structurally similar soluble electron acceptor, [Os(N,Nʹ-dimethyl-2,2ʹ-biimidazole)_3_]^2+/3+^, with a close standard redox potential (−0.216 V) was studied [3]. The maximum catalytic oxidation current with the soluble complex was observed also at pH 9. At pH-values lower than pH 5.8 no catalytic signal was detected. Thus, the pH-dependencies of PaoABC in the osmium complex containing polymer and in the solution look similar. This indicates that the electron transfer between osmium redox complex and enzyme plays an essential role and is enabled at pH higher than 6.

### 3.2. pH-Dependence at Different Ionic Strengths for PaoABC Immobilized in Osmium Complex Containing Redox Polymer

Interestingly, the pH-optimum of PaoABC with osmium redox complex is very close to the calculated pI-value of FAD-containing subunit of PaoABC [3]. Taking into account that the osmium containing polymer carries positively charged redox moieties, electrostatic interactions should play an important role. If electrostatic interactions are essential for the electron transfer reaction, increasing salt concentration must have a great effect on the current generation due to shielding of charges making electrostatic interactions weaker and thus electron transfer slower. This is indeed observed for measurements in alkaline region.

**Figure 3:**
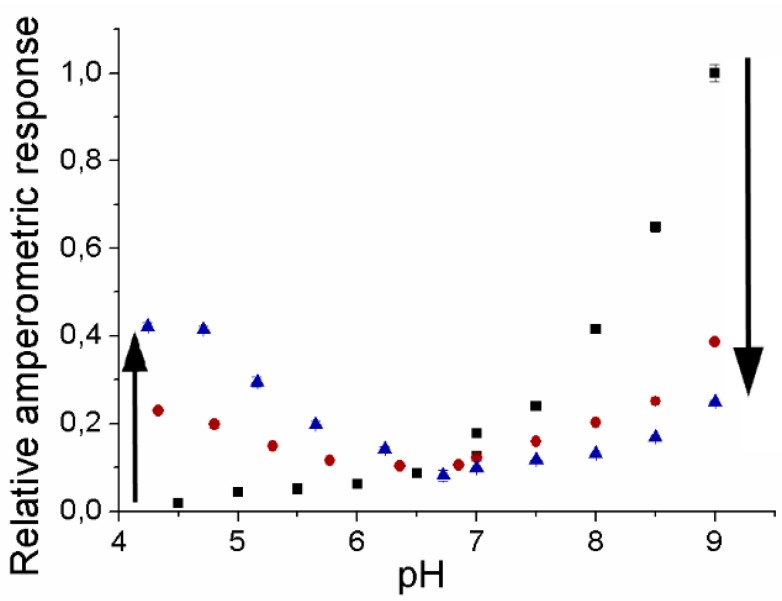
The effect of ionic strength on the amperometric response of PaoABC/Redoxpolymer-sensor towards 5 µM benzaldehyde in citrate-phosphate buffer (pH 4–7), in Tris buffer (pH 7-9). ■—in the absence of KCl, and ●—with 0.5 M KCl and ▲—1 M KCl, working potential 0 mV (*vs*. Ag/AgCl, 1 M KCl), n = 3.

The pH-dependence of the amperometric responses for PaoABC immobilized in osmium redox complex modified polymer towards 5 µM benzaldehyde depends on ionic strength (Figure 3). The variation of ionic strength was achieved by the adjustment of the ionic strength of buffers to 50 mM, and the further addition of 500 mM KCl and 1 M KCl.

The addition of KCl results in a decrease of the response of the PaoABC-biosensor in the alkaline region and increase of response in acidic solutions from no response up to 40% of the highest amperometric response at pH 9, while the response around pH 6.5 is low and not influenced (Figure 3).

Also, with the soluble osmium complex a drop of activity has been observed recently in the basic pH-range indicating a minor influence of the pH on the polymer [3]. This effect appears most probably because of the weakening of electrostatic attraction when increasing the salt concentration. However, the activation effect of the wired PaoABC at pH 4 was not observed in the previously published solution experiments [2] and may be attributed to the 30 mV higher reduction potential of the bound osmium complex compared to the free and/or to the hydrogel matrix. These results show the importance of the electrostatic effect on the electron transfer between positively charged osmium redox moieties and prove that wired PaoABC is active either at alkaline and acidic conditions.

### 3.3. Biosensor for Benzaldehyde

The potential of the wired PaoABC for biosensing was exploited for two cases, a benzaldehyde biosensor and a coupled enzyme approach for a GABA-biosensor.

**Figure A5:**
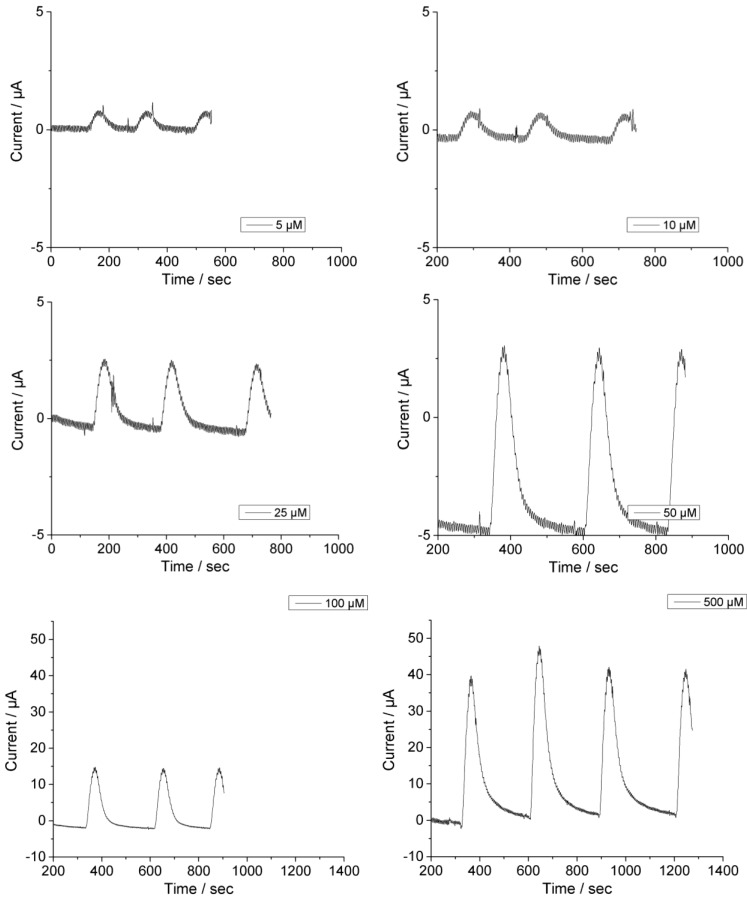
The responses of the PaoABC/Redox-polymer biosensor on 5–500 µM benzaldehyde, 50 mM Tris, pH 9.0, working potential 0 mV.

For the benzaldehyde biosensor PaoABC was immobilized in the osmium containing polymer on a carbon screen printed electrode and crosslinked with PEGDGE. The sensor was placed in a flow system. A catalytic oxidation current was observed when the benzaldehyde sample was flushed through the system (Appendix Figure A5). The signal appeared rapidly and reached the maximum within a few seconds. The signal returned quickly to the baseline when the flowing solution changed to buffer.

In order to increase the sensitivity the amount of redox polymer and enzyme was enlarged by step-by-step immobilization of enzyme/polymer layers one on the top of the previous layer similar to the strategy described recently [14].

**Figure 4:**
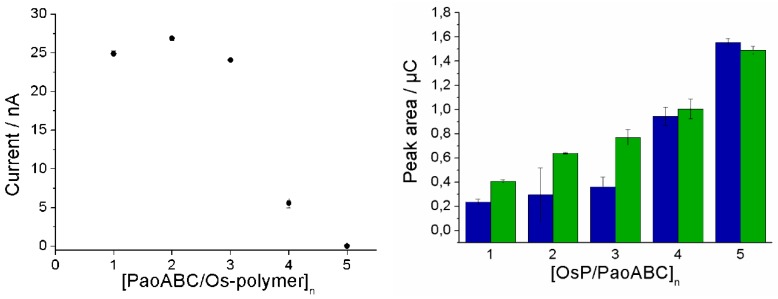
Effect of loading the 1mm diameter screen-printed working electrode with PaoABC/Redoxpolymer **Left**: The amperometric responses of PaoABC/Redoxpolymer-biosensor towards 200 µM benzaldehyde as a function of the number of successive immobilization steps, 50 mM Tris buffer, pH 8.0, working potential 0 mV, n = 3. **Right**: The anodic (blue) and cathodic (green) peak areas obtained from CVs in the absence of substrate for the electrodes with different number of layers. For the first layer, 2.5 µL PaoABC, 2.5 µL Os-Polymer and 1 µL PEGDGE were deposited. The next layers were prepared by deposition of each 1 µL PaoABC, 1 µL Os-P, and 1 µL PEGDGE.

Figure 4 shows that the charge increased with increasing polymer layers and thus reveals growing loading with electroactive osmium complexes. In contrast, the amperometric catalytic response towards 200 µM benzaldehyde showed only a minor change when the first layer was covered with a second layer. Thus, already with two layers maximum sensitivity was obtained (further layers dropped the sensitivity). The biosensor with four layers showed in spite of the higher redox polymer content decreased amperometric responses most probably due to the increased resistance for substrate diffusion.

**Figure 5:**
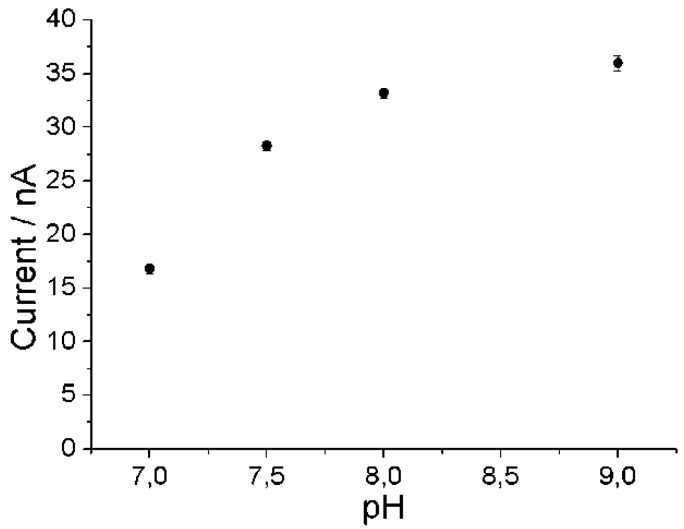
Amperometric response in a flow system cell with PaoABC/Redox-polymer-modified screen-printed carbon electrode towards 200 µM benzaldehyde, flow rate 300 µL/min, 50 mM Tris, 100 mM KCl, working potential 0 mV, n = 3.

Figure 5 shows that the response of the biosensor with two layers of redox polymer shows also an optimum at alkaline pH in 50 mM Tris-buffer, 100 mM KCl. Despite the effect of KCl on the sensor sensitivity at alkaline pH, 100 mM KCl was added for sake of reference to electrode stability. The curve is much broader than the initial pH-dependence with a lower enzyme loading. This is a typical behavior of enzyme sensors with diffusion-limited response.

**Figure 6:**
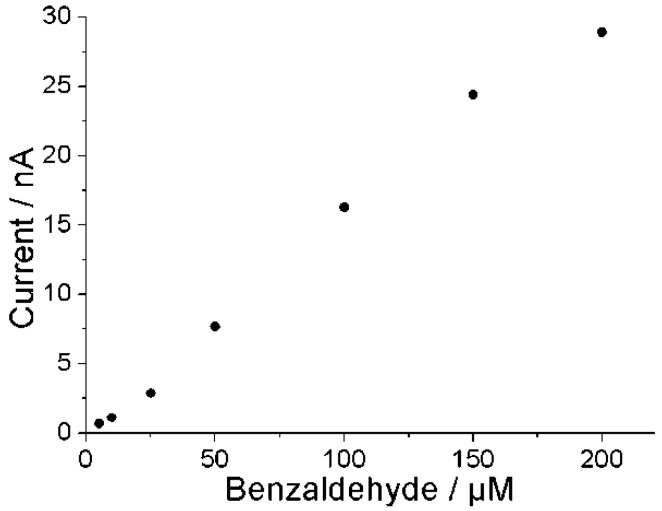
The calibration curve of PaoABC/Redox-polymer-modified screen-printed carbon electrode in a flow cell, flow rate 300 µL/min, 50 mM Tris, 100 mM KCl, pH 9.0, working potential 0 mV, n = 3 measurements per concentration with one electrode.

Under optimum conditions for the PaoABC-biosensor the sensor response in the concentration range between 10 and 150 µM increases with a slope of 0.15 nA/µM benzaldehyde with the detection limit of 5 µM (signal to noise ratio 3:1) as shown in Figure 6 for repetitive measurements made with one biosensor. Using the geometric area of the working electrode surface, the estimated sensitivity is here 19 nA/cm^2^ per µM benzaldehyde. Addition of benzaldehyde quickly changes the oxidation current with an average response time of 30 s and a total measuring time of 3 min.

**Figure 7:**
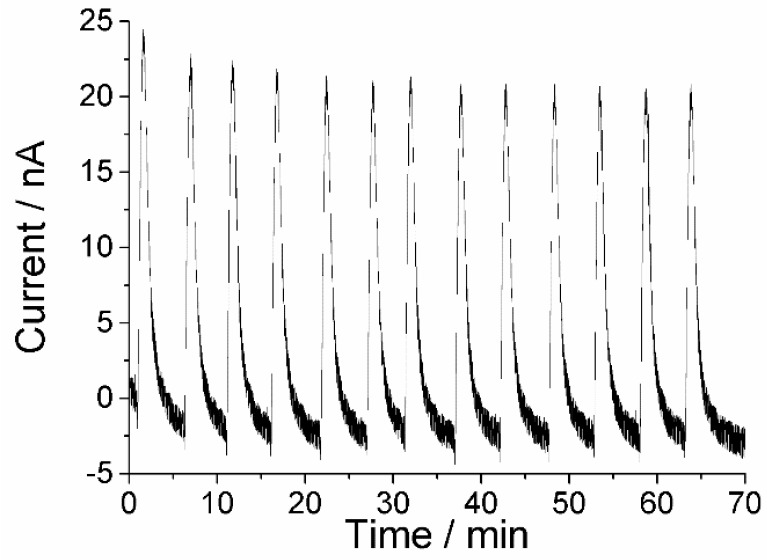
Response trace of the PaoABC/Redox-polymer-biosensor, for repetitive injections of 200 µM benzaldehyde, 50 mM Tris, pH 9.0, 100 mM KCl, working potential 0 mV.

The sensor response is repeatable (Figure 7) with 1.9% relative standard deviation in a series (n = 13, 200 µM benzaldehyde). The variation between three sensors is 14% for the measurement of 200 µM benzaldehyde. Sixty-eight percent of the initial sensitivity was retained after five days of measurements, when the sensor was stored refrigerated between the daily measurements.

In comparison to the previously published biosensor operating with soluble mediator [2], the novel developed biosensor shows a broader measuring range but higher detection limit. In summary, there are several advantages. The described system is reagentless due to the mediator covalently attached to the hydrogel, the measurement is much faster and the flow-cell arrangement allows for the automation of the detection process. Furthermore, in a series of repetitive measurements, it shows two times smaller coefficient of variation than in [2].

### 3.4. Biosensor for GABA

The γ-aminobutyric acid (GABA) is one of the major neurotransmitters in the central nervous system and has inhibitory function. Alteration in the concentration relative to other neurotransmitters has been suggested to be involved in several neuropathological disorders, such as epilepsy, Huntington’s disease, and Parkinson's disease, and so its accurate and continuous monitoring is of great importance [30]. The concentrations might vary from lower µM to mM values. For example, concentrations of 20–70 µM GABA have been reported for rat brain slices [31]. Direct GABA measurements are difficult, because GABA belongs to non-electroactive neurotransmitters and has a low extinction coefficient. In an enzyme based assay, a crude enzyme extract called GABAse, which is a mixture of a GABA-T and succinic semialdehyde dehydrogenase (SSDH) is applied for spectrophotometric measurement [32].

In a previously reported study, we observed catalytic activity PaoABC towards oxidation of succinic semialdehyde [2]. Here, we considered utilizing this activity in biosensors together with GABA-T, which catalyzes the transfer of an amino group from GABA to 2-oxoglutarate and formation of succinic semialdehyde and glutamate:

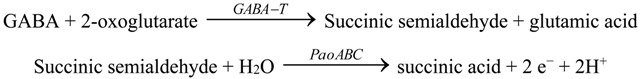


**Figure 8:**
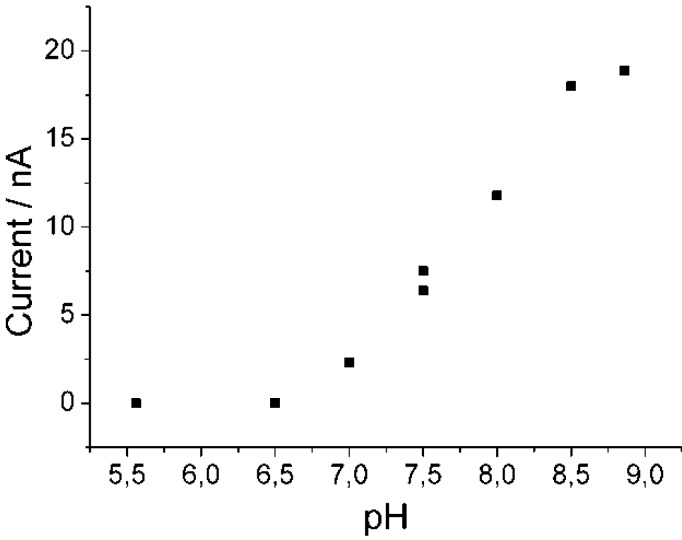
pH-Dependence of amperometric response of this biosensor towards 200 µM GABA, 100 mM phosphate buffer pH 5.5–7.5, 50 mM Tris buffer pH 7.5–9.0, 2 mM 2-oxoglutarate, 0 mV (*vs*. Ag|AgCl|1 M KCl).

The principle of operation of the novel system is based on the consecutive reaction of GABA-T and PaoABC. Succinic semialdehyde formed by GABA-T from GABA is oxidized by PaoABC to the respective dicarboxylic acid. GABA-T has therefore been expressed in *E.coli* and purified according to [27,28]. In the initial experiments (not shown), PaoABC and the purified GABA-T were coimmobilized in PVA and fixed onto a carbon electrode similar to the previously described system [2]. The electrochemical communication between PaoABC and an electrode was realized by the soluble mediator ferricyanide. The pH-profile of the biosensor reactions revealed that GABA measurements required a basic pH, similar to the physiological pH-optimum of GABA-T, while the pH-optimum for succinic semialdehyde oxidation had an optimum at acidic conditions, reflecting the pH-optimum for the PaoABC reaction with ferricyanide. The sensitivity for GABA was low because of the discrepancy of pH-optima between PaoABC with ferricyanide and GABA-T. Therefore, another mediator was selected, due to the fact that the pH-optimum of PaoABC can be tuned by the utilized mediator for a GABA-biosensor. GABA-T and PaoABC were coimmobilized in the Os-polymer and crosslinked with PEGDGE on spectroscopic graphite electrode, because PaoABC immobilized with this mediator obeys pH optimum for benzaldehyde oxidation in the desired basic pH range [3]. Figure 8 shows that the highest response is indeed generated at pH 9. This pH-value is similar to the optimum of PaoABC in osmium containing polymer and to the pH-optimum of GABA-T.

**Figure 9:**
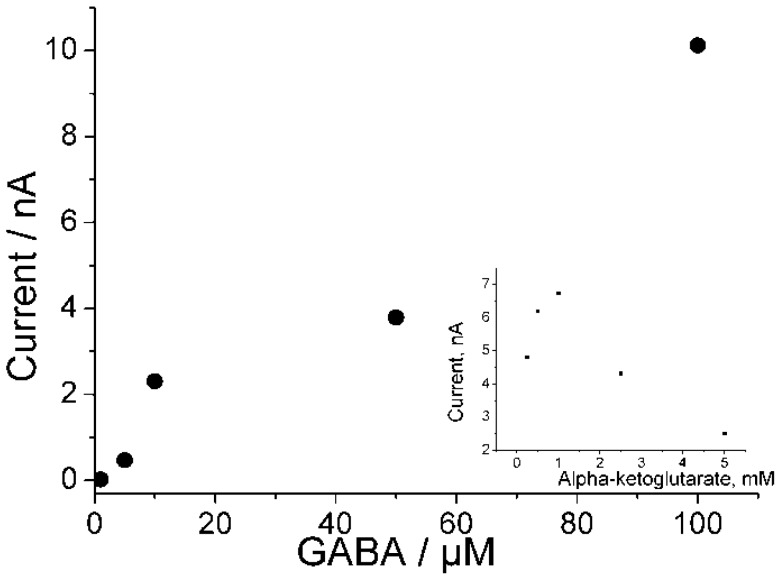
Dependence of the current response of the GABA-T/PaoABC/redox polymer-biosensor on the GABA concentration. Measurements were performed in 100 mM phosphate buffer pH 8, containing 2 mM 2-oxoglutarate. Applied potential 0 V (Ag|AgCl|1 M KCl). Inset shows the dependence of amperometric response on the 2-oxoglutarate concentration.

The addition of 5 µM GABA caused a current response of already 0.6 nA, *i.e*., 76 nA/cm^2^. The current response of the GABA-T/PaoABC/Os-polymer biosensor depends on the GABA concentration as is shown in Figure 9 for a measurement at pH 8, where the current response increased until 100 µM GABA with this biosensor. The response to GABA addition was maximal in the presence of 1 mM of the cosubstrate 2-oxoglutarate. These results are a first experimental proof of a biosensor for the inhibitory neurotransmitter GABA based on coimmobilization of GABA-T and PaoABC. Biosensors for GABA are only very rarely described. Basically, only two ways to determine the GABA concentration with biosensors were reported. The first way is to use GABA-T and detect the glutamate concentration, for example, by applying glutamate oxidase and peroxidase [33]. This method has a better detection limit then the new PaoABC/GABA-T biosensor. However, it suffers from response to glutamate, which is another amino acid neurotransmitter and can be present in varying concentrations in the sample, and by a cosubstrate dependence. The second approach utilizes the commercially available product called “GABAse” [34] which appears as a non-defined mixture of GABA-T and succinic semialdehyde dehydrogenase (SSDH). In this case, the transamination to succinic semialdehyde is followed by the SSDH conversion measured also through cosubstrate concentrations. Unfortunately, it is not possible to vary the single enzyme concentrations in GABAse. The here described biosensor provides an opportunity to overcome these difficulties by using PaoABC and isolated GABA-T coimmobilized in osmium complex bound polymer, and represents a new principle of bioelectrochemical GABA-detection.

## 4. Conclusions

PaoABC aldehyde oxidoreductase was successfully immobilized in osmium containing redox polymer on spectrographic graphite electrode and on the carbon screen printed electrodes for a fluidic sensor approach. We demonstrate that the osmium containing redox polymer is a suitable redox mediator for PaoABC and their combination creates a stable and sensitive bioelectrochemical system operating reproducible as a biosensor for benzaldehyde at low potential. The described system has two certain advantages for biosensor approach, the lower working potential preventing the unspecific oxidation on the electrode and “reagentless” interface of the system where the mediator is coimmobilized with an enzyme and need not to be added to the solution again. Due to the absence of leachable components, the biosensor could be implemented in a flow cell permitting the automation of the measuring process, decrease of the measuring time and increased repeatability in comparison to the conventional approach with a soluble mediator [3]. This will be valuable for example for determination of benzaldehyde impurities in benzyl alcohol, a reagent which is commonly used as preservative in pharmaceutical formulations.

**Figure A6:**
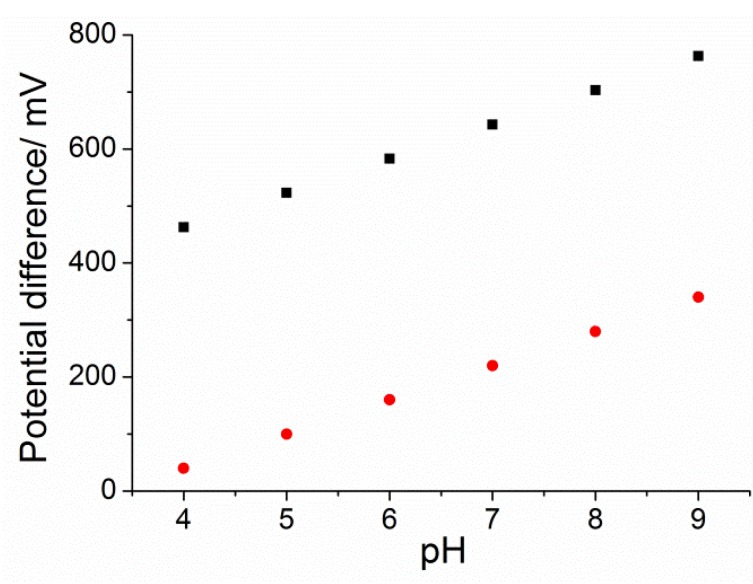
The estimated driving force between the PaoABC redox site with the most positive potential and osmium complex containing redox polymer (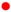) or ferricyanide (■).

Beyond the benzaldehyde biosensor function, we could show that the electron transfer between PaoABC and a redox polymer with a positively charged osmium redox complex happens at basic conditions (pH 9) with almost no activity at pH 4.5 and below. This behaviour is in full accordance with experiments done with a soluble osmium redox complex [3], but different then the previously described [1,2] pH-dependence with negatively charged ferricyanide having the pH-optimum at pH 4–5 with a mirror like shape of pH-dependence. Both mediator complexes contain a central metal atom, osmium or iron, and six occupied coordination sites. [Fe(CN)_6_]^4−/3−^ has the standard potential +190 mV which is about 400 mV higher than the standard potential of the polymer bound osmium complex, PVP-[Os(N,Nʹ-dialkylated-2,2ʹ-biimidazole)_3_]^2+/3+^. The electrochemical half reactions of the polymer bound osmium complex and ferricyanide [35] are almost pH-independent (less than 10 mV/pH) under the conditions of these experiments. On the contrary, the potentials of PaoABC redox cofactors determined in noncatalytic direct voltammetry experiment are pH-dependent and show about 60 mV shift per pH-unit [2] (see Appendix Figure A6 for pH dependence and respective driving force). The variation of ionic strength showed that the contribution of electrostatic interactions is essential and the polymer contribution to this effect is low. The behavior is similar to the interaction between the similar soluble osmium complex and PaoABC in homogeneous solution [3]. Therefore, we conclude also here that the redox complex interacts with the FAD-containing subunit PaoB. Moreover, at higher ionic strength the osmium containing redox polymer immobilized PaoABC can be activated also at lower pH and reveals a significant activity at pH 4.5 for benzaldehyde detection with smaller activity at pH 6–7. This effect looks very promising particularly for pH-responsive systems, where normally pH-responsive polymers are exploited, and which has gained increasing interest in last decades [15]. The system developed in this paper shows that PaoABC can play the role of a pH-responsive and catalytic element.

The finding that PaoABC has a pH optimum at basic conditions being immobilized in osmium complex modified hydrogel is important for its application in a novel GABA-biosensor, where PaoABC and GABA-T were entrapped in osmium containing polymer. The choice of this immobilized redox mediator caused an elimination of pH-optimum discrepancy resulting in lower detection limit and avoids the need to add an electron mediator. Detailed optimization for a certain sample is therefore the subject of further studies. Our approach looks promising and can be transferred in the future to a microelectrode to perform measurements in body tissue.

## Appendix

### Effect of Working Potential

A working potential of 0 mV *versus* Ag/AgCl/1 M KCl is sufficiently high to achieve high catalytic oxidation current (Figure A1A,B).

### Electrochemical Behavior of Redox Polymer with Bound Osmium Complexes

The polymer bound osmium complex is poly (4-vinylpyridine)-[osmium-(N,N′-methylated-2,2′-biimidalzole)_3_]^2+/3+^. The buffer solutions were prepared in the range of pH 5–8 with KCl—content from 0 M to 1 M. Cyclic voltammograms of the polymer bound osmium complex on a spectrographic graphite electrode were measured in these buffers, and the peak current and cathodic and anodic peak potentials for the transformation of Os^2+/3+^ in the bound complex were evaluated. There was no drastic change of electrochemical parameters of the redox polymer (Figure A2, Figure A3 and Figure A4) in solutions of different pH and ionic strength.

### The Driving Force Estimation

Using non-catalytic voltammetry of PaoABC deposited on highly oriented pyrolytic graphite three distinguished reduction potentials were obtained. The values are at pH 6.5, −643 mV, −517 mV and −433 mV (*vs.* Ag|AgCl|1 M KCl) and are increasing 59 mV/pH [2]. The highest value was taken for the calculation of the driving force (Figure A6). The potential is comparable to values found for FAD of XOD from cow milk and close to XDH determined by PFV and EPR potentiometry. We therefore assigned the highest peak also to FAD. In the previous paper it was also described that addition of benzaldehyde did not show any catalytic response unless a mediator has been added to the solution [2]. The slope of 59 mV/pH together with the lack of a semiquinone intermediate [1] supports the two proton/ two electron process.
